# Complementary feeding practices, dietary diversity, and nutrient composition of complementary foods of children 6–24 months old in Jimma Zone, Southwest Ethiopia

**DOI:** 10.1186/s41043-019-0172-6

**Published:** 2019-06-03

**Authors:** Sirawdink Fikreyesus Forsido, Nejat Kiyak, Tefera Belachew, Oliver Hensel

**Affiliations:** 1 Department of Agricultural and Biosystems Engineering, Faculty of Organic Agricultural Sciences, University of Kassel, Witzenhausen, Germany; 2 Department of Population and Family Health, Nutrition Unit, College of Public Health and Medical Sciences, Jimma University, Jimma, Ethiopia; 3 Department of Post-Harvest Management, College of Agriculture and Veterinary Medicine, Jimma University, Jimma, Ethiopia

**Keywords:** Dietary diversity, Nutrient adequacy, Complementary foods, Proximate composition

## Abstract

**Background:**

Mothers and caregivers typically feed infants according to their culture, purchase power and level of awareness with no due diligence to nutritional quality of the diet. Scientific evidence on nutritional adequacy of predominant complementary foods is critical for planning and prioritising interventions. The purpose of the current study was to evaluate the quality of complementary foods and the optimality of complementary feeding practices in Southwest Ethiopia.

**Methods:**

In this cross-sectional study, a stratified multistage sampling procedure was used to sample 433 children, 6–24 months old. A semi-structured questionnaire was used to collect demographic, socio-economic and dietary data. Dietary diversity score was measured using a 24-h dietary recall. Six customary complementary food types were assayed for proximate composition, energy and mineral density using standard methods. Adequacy of the complementary foods in nutrients for complementary feeding purposes was assessed as a ratio between actual composition and recommended composition of complementary foods.

**Results:**

Only 16.1% of the children get the minimum dietary diversity. The children were reported to be fed with cereals & grains (68.8%), discretionary calories (53.6%), protein-rich foods (44.6%), oils and fat (40.5%), vegetables (38.5%), dairy products (17.9%) and fruits (28.1%). The sampled foods contained 4.3–24.4%, 0.9–8.5%, 8.2–11.9%, 27.9–162.6 Kcal/100 g, 168.4–250.4 mg/100 g, 1.8–4.1 mg/100 g and 22.5–42.4 mg/100 g of total carbohydrate, crude fat, protein, energy content, calcium, zinc and iron, respectively. All the complementary food samples predominantly fed to children were not composed of adequate protein, fat, carbohydrate, energy and calcium as recommended for complementary feeding purposes. However, most of the complementary foods are composed of adequate iron and zinc.

**Conclusions:**

The nutrient density and diversity of complementary foods of 6–24-month-old children in the study area were found to be sub-optimal. Upgrading the nutritional composition of the starchy complementary foods should be of highest priority to improve nutrition of the infants and young children.

## Background

Complementary feeding refers to supplementing breastfeeding with feeding children aged between 6 and 24 months with a wide range of foods [10]. The period between 6 and 24 months of age is a time of nutritional vulnerability because during this period, nutrients especially micronutrients and energy obtained only from breast milk will not be sufficient to meet the requirements of the child [22]. Ensuring adequate nutrition during the period between 6 and 24 months of age is a major global health priority [10].

Among the immediate causes of undernutrition among children is consumption of too few nutrients [5]. In most low-income countries, including Ethiopia, the beginning of growth faltering coincides with the start of complementary feeding; age-specific malnutrition rates generally increase until about 24 months of age and then level off [20]. The sharp rise in the occurrence of stunting in young children from the age of 6 months is usually associated with suboptimal complementary feeding practices [9]. As children younger than 24 months old do not consume a sufficient amount of food to cover the high nutrient needs for growth and development, food given to them should be of high nutrient density [10].

Theoretically, infants should receive the most nutrient-dense diet in the family. Infants in low-income countries, however, are typically fed with nutrient-poor foods like thin porridges [10]. Complementary foods should contain high-biological value protein, furthermore, vitamins and minerals [25].

The National Nutrition Strategy (NNS) of Ethiopia gives considerable emphasis for nutrition of children younger than 2 years old in particular as nutrition received during this period influences how the children develop, grow and learn now or later. There is no documented evidence of overall complementary feeding practices and adequacy of the complementary foods in nutrients in Jimma Zone. This information is critically needed to be able to judge and plan the mechanisms to upgrade traditional diets. Therefore, this study aimed to evaluate the complementary feeding practices, dietary diversity and nutrient adequacy of complementary foods of children 6–24 months old in Jimma Zone, Southwest Ethiopia.

## Methods

### Area and subjects

This study was conducted in Jimma Zone, Southwest Ethiopia. The study area is year-round green but unfortunately characterised by household food insecurity [4]. Three districts were purposively selected based on their agricultural production; Omo Nada, Dedo and Mana are cereal, vegetable and cash crop producer areas, respectively.

This study is a component of a more prominent cross-sectional study which assessed the nutritional status and associated factors among children younger than 2 years old. The study population for the original research included all children younger than 2 years old in the study area. A multistage stratified sampling procedure was used to sample 558 children who were 0–24 months old [14]. For the current study, only those children 6–24 months old were included.

### Data collection

Data were collected from mothers or caregivers of the infants and children using face-to-face interviews using a semi-structured questionnaire.

### Variables

The variables were categorised as dependent and independent variables. The dependent variable was the dietary diversity score of the 6–24-month-old children. The independent variables included several socio-economic and demographic factors like family composition, household size, educational level attained by mothers and fathers, the occupation of mothers and fathers, the wealth of the household and education or training received on health and nutrition. Additionally, the infant- and young child-feeding (IYCF) practices were also assessed.

### Measurements

#### Diet diversity

A single 24-h dietary recall was used to obtain data on dietary diversity. Dietary diversity was assessed with a scale of seven food groups namely cereals and grains, vegetables, fruits, dairy products, oil and fat, protein-rich foods and discretionary calorie foods. Dietary diversity score (DDS) was found to be optimal when a child is fed greater than four food groups per day [23].

#### Nutrient composition of complementary foods

First, the complementary foods fed to 6–24-month-old children were identified from the questionnaires. Complementary food samples (25 g) were collected from 217 households. The collected food samples were labelled and stored at − 18 °C and eventually transferred to Jimma on the same date of collection. Based on the ingredients used for making the complementary foods, 6 assays were identified. The gross sample was then reduced in size and homogenised to create the laboratory sample [19]. The samples were then analysed for proximate composition (protein, fat, carbohydrate, moisture, ash and fibre), energy content, mineral (iron, zinc, calcium and phosphorous) and anti-nutritional factors (phytate and tannin) following the respective standard methods of analysis [3].

#### Nutrient adequacy of the complementary foods

Adequacy of the complementary foods in nutrients for complementary feeding purposes was assessed as a ratio between actual composition and recommended composition of complementary foods [13].

### Statistical analysis

The data were analysed using Statistical Package for Social Sciences software version 20 (SPSS Inc., Chicago, IL, USA). Descriptive statistics such as percentages were calculated. Bivariate analysis was conducted for the dietary diversity data. *P* values of less than 0.05 were regarded as statistically significant.

## Results

### Characteristics of the sample

Table 1 presents the socio-demographic characteristics of the respondents. Fifty four percent of the children in this study were male and 46% were female. Four hundred thirty-three out of 558 children included in the study were aged 6–24 months old. The larger part of the participants 372 (66.7%) were rural residents. A majority of the participants 516 (92.5%) was Muslims in religion. More than half of the children 306 (54.8%) were third and above in their birth order. Additionally, majority of the mothers 360 (64.5%) were aged 20–29 years old. Regarding literacy, larger part of the participants 336 (60.2%) did not attend formal education. A large proportion of the mothers 436 (78.1%) were housewives.

**Table 1:** Description of the study participants (Jimma Zone, Southwest Ethiopia, 2014)

Variable	Frequency (*N* = 558)	Percentage (%)
Sex of index child		
Male	302	54.1
Female	256	45.9
Age of index child (months)		
0–5	125	22.4
6–11	167	29.9
12–24	266	47.7
Mean	11.41 ± 6.5	
Residence		
Urban	186	33.3
Rural	372	66.7
Religion		
Muslim	516	92.5
Orthodox	33	5.9
Protestant	9	1.6
Birth order of index child		
First	128	22.9
Second	124	22.2
Third & above	306	54.8
Age of the mother (years)		
15–19	26	4.7
20–29	360	64.5
30–39	161	28.9
40–49	11	2.0
Mean	26.6 + 5.1 years	
Maternal education		
Formal education	222	39.8
No formal education	336	60.2
Maternal occupation		
Housewife	436	78.1
Merchant	83	14.9
Other^(a)^	39	7.0

^(a)^Daily labourer, government employee, farmer, other

### Complementary feeding practices

Table 2 shows the infant- and young child-feeding practices in the study area. The majority (88.9%) of the children were exclusively breastfed, and 75.6% were breastfed up to the age of 2 years. Both early and late initiation of additional food was practised extensively in the study area, but most (82.9%) of the mothers started to give complementary food to their children just at 6 months. However, nearly half of the mothers (53.8%) do not prepare any particular complementary food other than the typical family dish while the rest make some other additional foods of which gruel or *Atmit* is the predominant one. At the same point, almost all (91.6%) of the mothers do not prepare any particular food to their children during sickness or recovery from disease. The study also signified that 96.7% of the mothers feed their children 3–4 times a day.

**Table 2:** Complementary feeding practices of children 6–24 months old in three districts of Jimma Zone, Southwest Ethiopia, from March to May 2014

Variables	Number	Percent
Exclusive breastfeeding^a^		
Yes	496	88.9
No	62	11.1
Continued breastfeeding ≥ 2 years old^a^		
Yes	422	75.6
No	136	24.4
Time of initiation of complementary feeding		
Before 6 months	49	11.6
Just at 6 months	350	82.9
After 6 months	23	5.5
Preparation of special additional food		
Yes	258	46.2
No	300	53.8
Particular food during sickness or recovery		
Yes	47	8.4
No	511	91.6
Feeding frequency		
> 2 times a day	39	9.2
3–4 times a day	326	76.9
3–4 times + 1–2 snack	59	13.9
Dietary diversity score		
< 4 food groups per day	303	54.3
> 4 food groups per day	255	45.7
Food groups fed to children^b^		
Cereals and grains	384	68.8
Fruits	157	28.1
Vegetables	215	38.5
Protein rich	249	44.6
Dairy products	100	17.9
Oil and fat	226	40.5
Discretionary calories	299	53.6

^a^Sample size = 558

^b^Percentages do not add up to 100% as more than one response is possible

Regarding dietary intake, two thirds (68.8%) of the study participants consumed cereal-based gruel (made of barley, oat, teff, wheat, sorghum). Nearly half (44.6%) of the study participants reported that they fed their children with protein-rich food before the survey and (53.6%) of the study subjects consumed discretionary calories in the previous 24 h. Fruits, vegetables and dairy products were consumed by 28.1%, 38.5% and 17.9% of the participants, respectively.

### Dietary diversity

Table 3 presents the dietary diversity of the study participants. Majority of the children (83.9%) did not get the minimum dietary diversity. Dietary diversity score was significantly (*P* < 0.05) influenced by the age and birth order of the child, maternal and paternal education, socio-economic status of the family and paternal occupation. Higher dietary diversity was scored among children aged 12–24 months old, whose birth order was first and whose parents were formally educated. Children whose fathers are not farmers and children living in homes with higher wealth status also had higher DDS.

**Table 3:** Distribution of child DDS by different variables in Jimma Zone, South West Ethiopia

Variables	MDDS		
	No *n* (%)	Yes *n* (%)	*χ* ^2^ (*P* value)
Child age group			
0–5 months	124 (99.2%)	1 (0.8%)	0.000
6–11 months	147 (88.0%)	20 (12.0%)	
12–24 months	197 (74.1%)	69 (25.9%)	
Birth order of the child			
First	99 (77.3%)	29 (22.7%)	0.007
Second	99 (79.8%)	25 (20.2%)	
Third and above	270 (88.2%)	36 (11.8%)	
Districts			
Mana	159 (85.5%)	27 (14.5%)	0.470
Omo Nada	158 (84.9%)	28 (15.1%)	
Dedo	151 (81.2%)	35 (18.8%)	
Maternal education			
Informal education	293 (87.2%)	43 (12.8%)	0.006
Formal education	175 (78.8%)	47 (21.2%)	
Paternal education			
Informal education	205 (87.2%)	30 (12.8%)	0.041
Formal education	263 (81.4%)	60 (18.6%)	
Maternal occupation			
Housewife	370 (84.9%)	66 (15.1%)	0.144
Other	98 (80.3%)	24 (19.7%)	
Paternal occupation			
Farming	296 (86.5%)	46 (13.5%)	0.021
Other	172 (79.6%)	44 (20.4%)	
Place of residence			
Rural	316 (84.9%)	56 (15.1%)	0.196
Urban	152 (81.7%)	34 (18.3%)	
Wealth of households			
Poor	162 (87.1%)	24 (12.7%)	0.009
Medium	165 (87.3%)	24 (12.7%)	
Rich	141 (77.0%)	42 (23.0%)	

### Nutrient composition of complementary foods

The macronutrient composition and energy contents of the complementary foods are presented in Table 4. The moisture, total carbohydrate, protein, crude fat, total ash, crude fibre and energy content of the sampled complementary foods ranged between 58.4 and 79.6%, 4.29 and 24.44%, 8.21 and 11.87%, 0.86 and 8.49%, 2.94 and 8.03%, 2.28 and 8.19% and 27.87 and 162.57 cal, respectively.

**Table 4:** The proximate composition and calorific value of sampled complementary foods (*Atmits*) in three districts of Jimma Zone, Southwest Ethiopia from March–May, 2014

*Atmit* types	Fibre (%)	Fat (%)	Ash (%)	Protein (%)	CHO (%)	MC (%)	Calorific value (Kcal/100 g)
C	2.28	1.33	5.34	8.21	24.44	58.4	142.57
CO	2.65*	0.86	3.78	10.49	10.02	72.2	89.78
CPO	6.99	1.27	8.03	8.4	4.29	79.6	27.87
CPS	8.19	2.49	4.9	10.77	5.58	67.8	87.81
CP	6.41	4.49	5.15	9.67	9.78	64.5	118.21
COPS	5.73	8.49	2.94	11.87	9.67	61.3	162.57
Codex standard^(a)^	< 5	10–25	< 3	15	60–75	< 5	400–425

*Calculated from EHNRI [12]

*C* cereal, *O* oilseed, *P* pulse, *S* spice

^(a)^ CODEX CAC/GL 08 [13]

On the other hand, Table 5 presents the micronutrient and anti-nutrient composition of complementary foods dominantly consumed by the children. The mineral content ranged between 168.41 and 250.4 mg/100 g, 1.78 and 4.14 mg/100 g, 22.48 and 42.39 mg/100 g and 225.56 and 317 mg/100 g for calcium, zinc, iron, and phosphorus, respectively. The anti-nutritional factor contents ranged between BDL (below detection limit) of 117.72 mg/100 g for phytate and 1.17–75.17 mg/100 g for tannin.

**Table 5:** The mineral and anti-nutritional factor content of sampled complementary foods (*Atmits*) in three districts of Jimma Zone, Southwest Ethiopia, from March to May, 2014

*Atmit* types	Iron mg/100 g	Zinc mg/100 g	Calcium mg/100 g	Phosphorous mg/100 g	Phytate mg/100 g	Tannin mg/100 g
C	30.34	1.78	177.57	245.32	70.68	1.17
CO	33.86	2.86	206.18	257.62	96.33	7.22
CPO	42.39	2.81	168.41	225.56	BDL	22.36
CPS	36.52	2.9	198.12	272.44	BDL	45.5
CP	29.2	3.03	178	317	117.72	19.67
COPS	22.48	4.14	250.4	259.48	–	75.17
Codex standard^(a)^	16	3.2	500	456		

*C* cereal, *O* oilseed, *P* pulse, *S* spice, *BDL* below detectable level

^(a)^CODEX CAC/GL 08 [13]

### Nutrient adequacy of complementary foods for complementary feeding purposes

Table 6 presents nutrients adequacy of the studied complementary foods for complementary feeding purposes. All of the foods did not contain adequate amounts of protein, fat, energy, zinc and calcium (values < 1. On the other hand, most of the diets provide sufficient quantities of ash and iron (values > 1). The requirements were not met for any of the nutrients.

**Table 6:** Adequacy of the complementary foods in nutrients for complementary feeding purposes

Gruel types	Protein	Fat	CHO	Fibre	Ash	Energy	Fe	Zn	Ca
C	0.55*	0.13	0.38	0.46	1.78	0.36	1.9	0.56	0.36
CO	0.7	0.09	0.16	0.53	1.26	0.22	2.12	0.89	0.41
CPO	0.56	0.13	0.07	1.4	2.68	0.07	2.65	0.88	0.34
CPS	0.72	0.25	0.09	1.64	1.63	0.22	2.28	0.91	0.4
CP	0.64	0.45	0.15	1.28	1.72	0.3	1.83	0.95	0.36
COPS	0.79	0.85	0.15	1.15	0.98	0.41	1.41	1.29	0.5

*This numbers are the ratio between actual composition and recommended composition of complementary foods [13]

## Discussion

### Complementary feeding practices

Studies in developing countries showed that both too early and a too late introduction of complementary food is familiar. Mothers in South Africa start complementary feeding within 2–3 months. In Uganda, 44.1% and 27% mothers began complementary feeding within 2–3 months in 1997 and 2005, respectively [2]. Our findings oppose these reports because for the majority of the children in the study area complementary feeding is initiated just at the appropriate time.

Contrary to the recommended practice of complementary feeding, the majority of the mothers do not prepare particular food during sickness or recovery. During an illness, the need for fluid often increases, so a child should be offered and encouraged to take more [6]. However, feeding frequency in the study area was optimal for children in the age group of 6–12 but not for those in the age group of 13–24 months. The recommended number of meals per day for a healthy breastfed baby should be 2–3 times at 6–8 months, 3–4 times at 9–11 months and 3–4 times with 1–2 additional nutritious snacks at 12–23 months of age [25].

The predominant complementary food fed to children in the study areas is gruel (a liquid drink made of cereals), locally named *Atmit*, ranking first in the all of the three districts. In Ethiopia, 70% of children aged 6–23 months, predominantly consume foods made of grains [7]. Similarly, a study conducted in Nigeria reported that the dominant food groups in the children’s diet were cereal/grains [2]. Nutritional problems are common among populations whose diets are predominantly based on starchy staples [21], and these plant-based foods are low in micronutrient contents, high in phytate and high in dietary fibre which inhibits the absorption of micronutrients [17].

### Dietary diversity

The results of the current study showed that nearly half of the children had sub-optimal dietary diversity. A survey of community-based production complementary foods in four regions of Ethiopia has reported that the highest mean diversity score of 3.4 was observed in Oromia region, followed by Tigray (2.9) Amhara (2.7) and SNNP (2.6) regions. In Oromia region, nearly half (49.4%) of the children had a suboptimal diet diversity [1].

According to our results, children from wealthier households consumed more diversified diets. The ability to produce sufficient food for one’s household at home and generate enough revenue to purchase foods on the market are ways that a family could achieve food security [16].

The results of this study also indicated that girls are fed with less diversified diet compared to their male counterparts. Male gender bias in the intra-household distribution of food and other resources has been reported from Ethiopia [15] indicating that girls are less favoured in the resource-constrained environments.

### Composition of diets

The complementary foods were predominantly made of starch-based cereals and hence of poor nutritional value, and do not satisfy the infant’s basic needs of protein because they have limited levels of protein both qualitatively and quantitatively. As well, macro- and micronutrients may be insufficient to maintain growth and development, this results in reduced nutritional status in children [25].

### Nutrient adequacy of the complementary foods for complementary feeding purposes

Complementary foods should contain the following amount of estimated macronutrients: moisture content of ≤ 5%, an ash content of ≤ 3%, a crude protein content of ≥ 15%, a crude fat content of 10–25%, a crude fibre content of ≤ 5% and a carbohydrate content of 64 ± 4%. Additionally, the energy content of 400–425 kcal/100 g, a calcium content of 500 mg/100 g, the iron content of 16 mg/100 g and zinc content of 3.2 mg/100 g are also required [13]. Children younger than 2 years old and living in developing countries should consume approximately 137–187 g/day, 206–281 g/day and 378–515 g/day of complementary foods at the age of 6–8 months, 9–11 months and 12–24 months, respectively to meet their energy needs [18]. The total nutrient and energy requirements of healthy breastfeeding infants have been established [11]. The average nutrient and energy that a given complementary food should provide are estimated by subtracting average nutrient and energy content of breast milk from the total nutrient and energy requirement at each age [24]. The complementary food samples were not adequate in nutrients for complementary feeding purposes.

Over and under-reporting of infant dietary intakes is among the reasons that made quantifying infants’ diets difficult. Parents tend to over-report intakes for they do not wish to be seen either to be under-feeding babies or on the one hand not being able to distinguish between food offered to young children and the amount consumed [8].

## Conclusions

The feeding practices of 6–24-month-old children in the study area were not satisfactory. Dietary diversity and macronutrient, energy and overall nutrient composition of the complementary foods were below the recommendations. The complementary foods were found to contain adequate amounts of iron. Improving the nutrient adequacy of the locally available customary diets through food processing techniques and a community-based nutritional education on optimal child feeding are recommended.
